# Genetic Impact of a Severe El Niño Event on Galápagos Marine Iguanas (*Amblyrhynchus cristatus*)

**DOI:** 10.1371/journal.pone.0001285

**Published:** 2007-12-12

**Authors:** Sebastian Steinfartz, Scott Glaberman, Deborah Lanterbecq, Cruz Marquez, Kornelia Rassmann, Adalgisa Caccone

**Affiliations:** 1 Department of Ecology and Evolutionary Biology, Yale Institute for Biospheric Studies, Yale University, New Haven, Connecticut, United States of America; 2 Yale Institute for Biospheric Studies, Yale University, New Haven, Connecticut, United States of America; 3 Charles Darwin Research Station, Puerto Ayora, Santa Cruz, Galápagos, Ecuador; 4 Achmühle, Eurasburg, Germany; University of Kent, United Kingdom

## Abstract

The El Niño-Southern Oscillation (ENSO) is a major source of climatic disturbance, impacting the dynamics of ecosystems worldwide. Recent models predict that human-generated rises in green-house gas levels will cause an increase in the strength and frequency of El Niño warming events in the next several decades, highlighting the need to understand the potential biological consequences of increased ENSO activity. Studies have focused on the ecological and demographic implications of El Niño in a range of organisms, but there have been few systematic attempts to measure the impact of these processes on genetic diversity in populations. Here, we evaluate whether the 1997–1998 El Niño altered the genetic composition of Galápagos marine iguana populations from eleven islands, some of which experienced mortality rates of up to 90% as a result of El Niño warming. Specifically, we measured the temporal variation in microsatellite allele frequencies and mitochondrial DNA diversity (mtDNA) in samples collected before (1991/1993) and after (2004) the El Niño event. Based on microsatellite data, only one island (Marchena) showed signatures of a genetic bottleneck, where the harmonic mean of the effective population size (*N_e_*) was estimated to be less than 50 individuals during the period between samplings. Substantial decreases in mtDNA variation between time points were observed in populations from just two islands (Marchena and Genovesa). Our results suggests that, for the majority of islands, a single, intense El Niño event did not reduce marine iguana populations to the point where substantial neutral genetic diversity was lost. In the case of Marchena, simultaneous changes to both nuclear and mitochondrial DNA variation may also be the result of a volcanic eruption on the island in 1991. Therefore, studies that seek to evaluate the genetic impact of El Niño must also consider the confounding or potentially synergistic effect of other environmental and biological forces shaping populations.

## Introduction

The El Niño-Southern Oscillation (ENSO) refers to a complex set of ocean-atmosphere interactions that take place throughout the Pacific basin [1]. El Niño events, which represent one phase of ENSO, are characterized by an accumulation of warm surface water in the central and eastern regions of the tropical Pacific. The resulting high sea-surface temperatures (SST) feed back on atmospheric circulation patterns worldwide, causing a range of environmental changes, from severe droughts in parts of Asia and the Western Pacific, to harsh winter conditions and flooding in North America [2]–[3].

During periods of acute El Niño warming, there is widespread mortality in aquatic organisms in the eastern Pacific [4]. The suppression of ocean upwelling and the rise in SST cause a marked decrease in primary productivity with ecological consequences for the entire marine food web [5]. Paleoclimate data depicts a 15,000 year history of El Niño, making it part of the natural evolutionary process of many populations and ecosystems. However, climate models suggest that the strength and frequency of El Niño events have recently increased, and will continue to do so, due to global warming [6]. In fact, the El Niño events of 1982–1983 and 1997–1998 were the strongest recorded in the last century and perhaps the last 400 years [7]–[8]. Thus, in the visible future, human activity may push environmental conditions to new extremes, and the capacity of populations to respond to these changes remains largely unknown.

**Figure 1:**
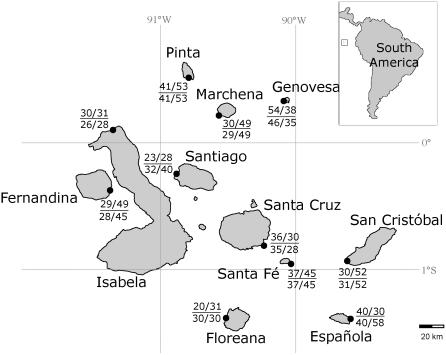
Map of the Galápagos archipelago in relation to South America and the Equatorial line (inlet). Localities are shown for the eleven islands sampled in 1991/1993 and 2004. For each population, sample sizes are labelled as follows: above the line are the number of individuals analyzed for 13 microsatellite loci from the 1991/1993 and 2004 samplings respectively; below the line are sample sizes used in mtDNA control region analysis from the 1991/1993 and 2004 samplings respectively.

The Galápagos archipelago (Figure 1) lies in the primary region of ENSO activity, experiencing the large increases in SST and rainfall that are characteristic of El Niño events [9]. In the absence of ocean upwelling, food abundance is low, causing starvation of many marine organisms [10]. During the most recent, severe El Niños, population crashes of 77% (1982–1983) and 65% (1997–1998) were recorded in the endemic Galápagos penguin [11], while nearly 100% of Galápagos fur seal yearlings and large males perished [12]. Perhaps the best-studied example of the impact of ENSO on natural populations is the Galápagos marine iguana (*Amblyrhynchus cristatus*), a species that is only found in the archipelago. Marine iguanas are known to inhabit the shoreline of all the major islands in Galápagos, where they forage almost exclusively on algae from intertidal and (nearshore) subtidal zones. The digestion of algae is made possible by a community of bacterial micro-symbionts that exist in the hindgut of the iguanas [13]. During severe El Niño events, when SST is elevated, the coastal environment is dominated by algae species that the iguanas cannot digest. On some islands, this has led to extreme undernourishment of iguanas, causing lowered body condition, increased stress hormone levels, significant changes in breeding behaviour, and mortality rates as high as 90% [14]–[17]. During La Niña, the other phase of ENSO, preferred algae are abundant and marine iguana populations have been shown to rebound quickly in terms of both census numbers and body condition [14].

Sharp population declines, or bottlenecks, like those seen during the recent El Niños, may translate into losses of genetic variation that can lead to increased rates of inbreeding and the fixation of deleterious alleles, and hinder the ability of populations to adapt to future changes in the environment [18]–[20]. In addition, there are a number of studies which have shown that decreased genetic diversity is associated with lowered immunocompetence, making populations more vulnerable to disease (see [21] for a recent example).

Although long-term demographic studies from several islands in Galápagos reveal the drastic effects of El Niño on marine iguana census numbers and reproductive behaviour, it is difficult to estimate the effective population size (*N_e_*) from such data, and methods based on demographic information tend to overestimate *N_e_*. Conversely, indirect methods of *N_e_* estimation based on genetic data have been shown to be particularly informative, making them an important component in evaluating the consequences of potential bottleneck events such as El Niño [22].

Only a few studies have examined the impact of ENSO on genetic diversity [23]–[25], but there have been no attempts to use genetic data to quantify *N_e_* in natural populations that have experienced a severe El Niño event. When samples from two or more generations are available, *N_e_* estimates based on the temporal variance of allele frequencies [26]–[27] have been shown to be the most reliable method for detecting a genetic bottleneck in both simulated [22] and empirical datasets [28]. This is because levels of genetic drift are inversely proportional to population size, causing rapid changes in allele frequencies in small populations, and the magnitude of these changes between two time points indicates the severity of the bottleneck [29].

In this study, we estimated the harmonic mean of *N_e_* for marine iguana populations from 11 islands between 1991/1993 and 2004, a period that covers the severe 1997–1998 El Niño. Effective population size estimates were derived from the temporal variance in allele frequencies of 13 microsatellite loci representing more than 800 individuals collected before (1991/1993) and at least one generation after (2004) the warming event. In order to corroborate our results, we also analysed the complete mitochondrial control region for the same populations and most individuals for signs of temporal genetic changes. The large number of individuals and genetic loci used in our analysis, combined with the temporal sampling design, makes this one of the most comprehensive studies to date trying to measure the short term genetic effects of a severe El Niño event on natural populations.

## Materials and Methods

### Sampling

Approximately 800 marine iguanas from 11 islands in the Galápagos archipelago were sampled at two different time points before (in 1991/1993) and after (in 2004) the 1997–1998 El Niño event (see Figure 1 and Table S1 for a detailed description of taken samples). Sample sizes for each population and time point ranged from 20–58 individuals. We sampled mainly semi-adult and adult marine iguanas with sex classes evenly distributed across samples. Approximately 1–2 ml of blood were taken from the caudal vein of each individual and placed in storage buffer (100 mM Tris, 100 mM EDTA, 2% SDS). Total genomic DNA was extracted using the QIAamp 96 DNA Blood Kit (Qiagen).

### Microsatellite analysis

Thirteen species-specific microsatellite loci were amplified in 806 individuals and scored for alleles as previously described [30]–[31]. We used the program NeESTIMATOR version 1.3 [32] and its implemented TM3 algorithm [33] to estimate effective population size (*N_e_*) by measuring the temporal variance of microsatellite loci allele frequencies between time points using a likelihood-based approach. For the analysis of temporal changes in allele frequencies for *N_e_* estimation, the program NeESTIMATOR requires the user to define a reference time point of a population (*generation 0*) and at least one subsequent generation (in our case *generation 1*). Island populations sampled in 1991/1993 were entered as *generation 0* and those sampled in 2004 as *generation 1*. Observed changes in allele frequencies and therefore estimates of *N_e_* only reflect changes that occurred from the reference point (*generation 0* in 1991/1993) to the subsequent point (*generation 1* in 2004). Since the TM3 program requires a maximum value for *N_e_*, we used the estimates of the maximum census size (*N_c_*) of a respective island population [14] as the upper bound of *N_e_*, as in most published studies, *N_c_* exceeds or equals *N_e_* [29]. *N_e_* was then estimated as the maximum likelihood value of 10,000 updates within that range.

F_ST_ differentiation between pre- and post-El Niño samplings was estimated with the program ARLEQUIN version 3.0 [34], including the test for significance (α = 0.05; see Table 1). Locus specific heterozygosity of samples from both time points was estimated with the program ARLEQUIN version 3.0 [34], and differences between time points for each island was tested with the non-parametric Wilcoxon signed-rank test using the Analyze-it® package for Microsoft Excel® for significance (α = 0.05; see Table 1 and Table S2).

**Table 1:** Summary analysis of the 13 microsatellite loci used in this study.

Island	Year of sampling	Heterozygosity±SD	Number of alleles±SD	F_ST_ between time samplings	Locus specific heterozygosity (*p* values)
Fernandina	1993	0.766±0.022	9.77±3.00	0.000	0.094
	2004	0.815±0.016	11.69±4.15		
San Cristóbal	1993	0.707±0.023	6.15±1.57	0.000	0.278
	2004	0.708±0.018	6.54±1.90		
Floreana	1993	0.810±0.025	7.85±2.97	0.008	0.787
	2004	0.755±0.022	9.62±4.09		
Genovesa	1991	0.701±0.018	6.92±1.85	0.000	0.588
	2004	0.705±0.021	6.46±2.26		
Marchena	1993	0.756±0.022	7.77±2.20	0.008*	0.032*
	2004	0.786±0.016	7.69±2.56		
Pinta	1993	0.647±0.021	5.85±1.07	0.003	0.305
	2004	0.637±0.018	6.31±1.49		
Santiago	1993	0.765±0.025	7.23±2.55	0.001	0.893
	2004	0.762±0.022	8.23±2.65		
Santa Cruz	1991	0.801±0.019	9.00±3.29	0.000	0.04*
	2004	0.814±0.020	9.08±3.59		
Española	1993	0.794±0.018	8.77±3.17	0.000	0.893
	2004	0.801±0.020	8.92±3.84		
Isabela	1993	0.762±0.022	9.38±2.96	0.000	0.893
	2004	0.769±0.021	9.38±2.75		
Santa Fé	1991/93	0.733±0.020	8.31±2.72	0.000	0.893
	2004	0.762±0.017	8.77±2.98		

Average heterozygosity and standard deviation (SD) and number of alleles with SD are shown for the pre- and post-El Niño time samplings of specific populations. Values of F_ST_ and locus specific heterozygosity values were calculated between time point samplings. In the last column, only the *p* values of the corresponding Wilcoxon signed-ranks test are provided for the locus specific differences in heterozygosity between time samplings of a specific population. The actual locus specific heterozygosity values can be found in Table S2. Significant differentiation (*p*<0.05) of F_ST_ values and locus specific heterozygosity values are marked with an asterisk (*).

### Mitochondrial analysis

Complete mitochondrial CR sequences (1183 base pairs) were generated for 838 marine iguanas (most of which were also analyzed for microsatellite loci). PCR was carried out on total genomic DNA using the primers IguanaCytb3 (5′-ACCAGTAGAACACCCMTTCATC-3′) and 12s1984 [35]. PCR was performed for 35 cycles with an annealing temperature of 57°C and an extension time of 90 seconds. PCR products were purified using the Qiaquick PCR Purification kit (Qiagen) and sequenced on an ABI3730 DNA analyzer (Applied Biosystems). All mtDNA haplotype sequences from this study were submitted to Genbank under accession numbers EU278255-EU278326. Haplotype diversity (Hd) and nucleotide diversity (π) values were calculated for each population and time point using the program DNASP version 4.10.9 [36]. Haplotype and nucleotide diversity were compared between time points for each population using the *t*-test described by Nei [37] with Hd and π variance values calculated in DNASP. F_ST_ values and their significance were calculated between time points for each population using ARLEQUIN version 3.0 [34] (Table 2). Coalescent simulations based on the *H*-test [38] were performed in DNASP (Table 2), and were used to determine whether populations from both time points had lower Hd values than expected under neutral evolution. For each population and time-point (e. g. Marchena in 2004), two separate simulations of 10,000 replicates were performed using the number of mutations across all sequences (*η*) and *θ* (2*N_ef_*μ; estimated from *k*, the average number of nucleotide differences between sequences). From this test, *p* values were obtained which actually reflect where the observed Hd value falls on the simulated distribution. Although the *H*-test was originally based on the number of segregating sites in a population (*S*), DNASP uses *η* in place of *S*. We chose to run the additional set of simulations with *θ* because it should be less sensitive to low frequency migrants, which can greatly elevate the value of *η*, and give a false signal of lower than expected Hd.

**Table 2:** Temporal analysis of mitochondrial control region data (1183 bp) for marine iguana populations from 11 islands.

Island	Year	No. of Haplotypes	F_ST_	95% CI Hd_sim_ (*η*)	*p* value	95% CI Hd_sim_ (*θ*)	*p* value
Española	1993	6	0.04018*	0.66154–0.93077	**0.0415**	0.42949–0.89615	0.2541
	2004	5		0.27526–0.79794	0.4285	0.22202–0.85360	0.3224
Fernandina	1993	18	0.02869*	0.68519–0.94444	0.81640	0.49206–0.91799	0.9701
	2004	23		0.72626–0.94343	0.99100	0.53232–0.91313	0.9999
Floreana	1993	6	0.08466*	0.62989–0.93103	**0.0305**	0.59080–0.92874	**0.0474**
	2004	10		0.65977–0.93563	0.4210	0.62989–0.93563	0.4792
Genovesa	1991	5	0.03575	0.55942–0.90242	0.0840	0.53720–0.91208	0.0989
	2004	4		0.56471–0.91092	**0.0061**	0.44202–0.90420	**0.0213**
Isabela	1993	6	−0.00697	0.56615–0.91692	0.2007	0.52923–0.92615	0.2096
	2004	5		0.52910–0.90212	0.0667	0.52116–0.92328	0.0663
Marchena	1993	4	0.44125*	0.47291–0.88177	0.2020	0.50493–0.92118	0.1318
	2004	2		0.44558–0.86224	**0.0019**	0.35969–0.88350	**0.0096**
Pinta	1993	4	−0.00848	0.23171–0.78659	0.0908	0.09512–0.82805	0.1738
	2004	5		0.27721–0.80189	0.1649	0.14441–0.84107	0.2207
San Cristóbal	1993	3	−0.01050	0.33333–0.82366	0.4161	0.37419–0.89677	0.2141
	2004	3		0.27903–0.80166	0.3789	0.34238–0.88235	0.1611
Santa Cruz	1991	1	0.00000	-	-	-	-
	2004	1		-	-	-	-
Santa Fé	1991/93	5	−0.01503	0.42492–0.86336	0.4256	0.45946–0.90390	0.2689
	2004	4		0.38384–0.84242	0.4221	0.48586–0.98707	0.1707
Santiago	1993	3	0.04572	0.58468–0.91532	0.0237	0.69556–0.94758	0.0055
	2004	2		0.55769–0.90000	0.0132	0.72692–0.94487	0.0005

Data from each island is separated by sampling year (1991/1993 or 2004). F_ST_ values represent differentiation between the same population at two time points. Significant F_ST_ values (*p*<0.05) are labelled with an asterisk. *H*-test simulations were performed in the program DnaSP using both the number of mutations across all sequences (*η*) and population parameter *θ* (see Material and Methods). The 95% confidence intervals (CI) of simulated Hd values are reported for both the *η*- and *θ*-based simulations. To the right of the 95% CI ranges are *p* values which signify the location of the observed Hd value on the simulated distribution. Populations where the observed Hd value falls within the lowest 5% of the simulated data have *p* values in bold. This signifies that a population has lower Hd than expected under a neutral coalescent model.

## Results

### Microsatellite analysis

The average heterozygosity of the 13 microsatellite loci calculated for each population and time point ranged from 0.637 for Pinta in 1993 to 0.815 for Fernandina in 2004. Island specific F_ST_ values calculated between pre- and post-El Niño samples were not significantly different from zero for most comparisons, but was 0.008 and significant (*p*<0.05) for Marchena (Table 1). The same level of F_ST_ differentiation (0.008) was also found between time point samplings on Floreana, but in this case it was not significant at the 5% level.

Based on the Wilcoxon signed-ranks test, locus specific heterozygosity was not significantly different (α = 0.05) between pre- and post-El Niño samples from a specific island, with the exception of Marchena and Santa Cruz (see *p*-values in Table 1 and the explicit locus specific comparisons in Table S2). The population on Marchena showed a significant decrease in locus specific heterozygosity (*p* = 0.032) from 1993 to 2004, while there was a significant increase (*p* = 0.04) on Santa Cruz from 1991 to 2004.

**Figure 2:**
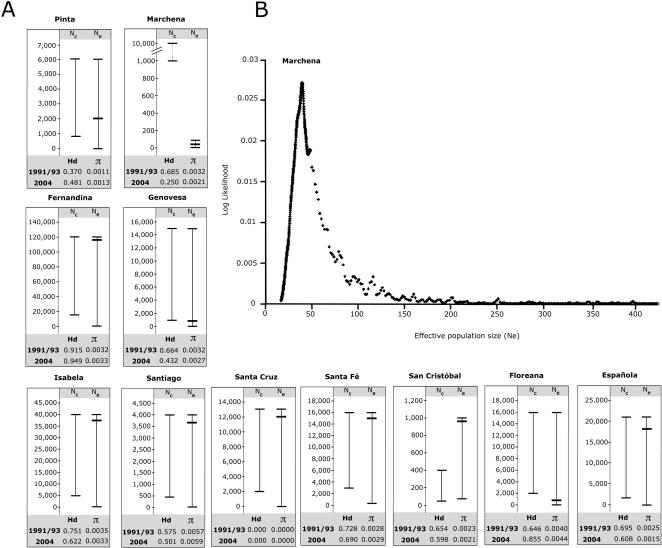
Illustration of estimated effective population size (*N_e_*) for Galápagos marine iguana populations. (A) Bar graphs showing the harmonic mean of the estimated effective population size (*N_e_*) for 11 marine iguana populations based on temporal variance of allele frequencies for 13 microsatellite loci between 1991/1993 and 2004. *N_c_* represents the census size range (with min. and max. lines) for each island population compiled from multiple field surveys [14]. The estimated harmonic mean of *N_e_* is represented by a single, bold, horizontal line within the broader 95% confidence interval (CI) of the respective estimate. MtDNA haplotype (Hd) and nucleotide (π) diversity values for each population are shown for both the 1991/1993 and 2004 samplings. Note: for all populations except Marchena, the estimated 95% CI of *N_e_* overlaps with the maximum estimate of *N_c_*. (B) Graphical display of the mean estimated *N_e_* indicative of the genetic bottleneck detected for marine iguanas on Marchena between 1993 and 2004. *N_e_* (x-axis) between 1993 and 2004 was estimated at 40 individuals (95% CI = 21–86 individuals) and is indicated by the peak of the log likelihood value (y-axis) at *N_e_* = 40. Please see Figure S1 for graphical displays of *N_e_* estimates for other populations and Materials and Methods for the approach to estimate *N_e_*.

Figure 2a shows the Bayesian estimates of *N_e_* and its associated confidence intervals (CI) obtained from microsatellite data using the temporal variance method. *N_e_* values for each population represent the harmonic mean of the effective population size over the time interval between pre- and post-El Niño samplings. A low *N_e_* value (<50 individuals) was only found for the population on Marchena, with a *N_e_* around 40 individuals and a narrow 95% CI of 21–86 individuals. For the remaining islands, *N_e_* estimates ranged from 770 and 783 individuals on the islands of Floreana and Genovesa respectively, to as high as 120,000 individuals on Fernandina. However, with the exception of Marchena, *N_e_* estimates from other islands had large CI values that overlapped with the minimum and maximum census size numbers of a specific island population [14] (Fig. 2a and Figure S1; see Material and Methods for details). Figure 2B depicts the low *N_e_* estimate of around 40 individuals (CI of 21–86 individuals) between 1993 and 2004 experienced by the population on Marchena (for comparison, see Figure S1 for plots of other populations). The large confidence intervals associated with *N_e_* estimates on the majority of islands shows that our study approach, given the number of individuals and microsatellite loci used, is not able to provide consistent estimates for large values of *N_e_*. This indicates that the effective population size must have remained fairly high on these islands during the time interval between samplings and that *N_e_* did not fall below the critical size of 50 individuals.

### Mitochondrial control region analysis

Mitochondrial control region (CR) data was generated for nearly the same set of individuals and populations used in the microsatellite analysis (see Table S1 for details). Among all the islands, the population from Marchena exhibited the largest decrease in haplotype diversity (Hd) and nucleotide diversity (π) between time points (Figure 2a; Table 2); however, only the difference in Hd was statistically significant (ΔHd = −0.435, *t* = 4.72, upper tail *p*<0.025; see Table S3). Although less severe, a loss of mitochondrial variability was also detected on Genovesa (ΔHd = −0.232, *t* = 2.11, upper tail *p*<0.025; see Table S3). All remaining populations did not exhibit significant changes in levels of mtDNA diversity (see Table S3 and Table S4). Mitochondrial CR F_ST_ values calculated between pre- and post-El Niño samples were rather low (<0.1; see Table 2) except for the population on Marchena (F_ST_ = 0.44125, *p*<0.05), and are therefore in line with the F_ST_ results from the microsatellite data.

Coalescent simulations of haplotype diversity (*H*-test) show that only Hd values for Marchena and Genovesa were significantly lower than expected under neutral evolution in samples from 2004 but not in those from 1991/1993, supporting the occurrence of some severe demographic event between time points (Table 2; see Material and Methods for *H*-test description). Several other populations do show lower than expected levels of haplotype diversity compared to neutral expectations, but only for the first time point (Española, Floreana) or for both time points (Santiago), which does not indicate that a genetic bottleneck occurred between time samplings. Only a single CR haplotype was found in samples collected on Santa Cruz in both 1991 and 2004, and therefore genetic diversity analyses and simulations could not be carried out for this island population.

## Discussion

### Study feasibility

The temporal sampling of genetic data can be a powerful means of estimating *N_e_* between two closely situated time points, as is the case in our study. However, there are certain requirements of such methods, particularly the temporal variance approach, which must be met in order to achieve valid *N_e_* estimates and a significant statistical power. Simulations based on the temporal variance method have shown that a bottleneck with only 34 surviving individuals can be detected with a probability of higher than 80% after one generation when using 12 moderately polymorphic microsatellite loci (with mean of 4.3 alleles per locus) and at least 30 individuals per population [22]. Since loci with higher levels of polymorphism increase statistical power [22], the use of 13 highly polymorphic microsatellite loci (with a mean of 8 alleles per locus; see Table 1) in our study improves our ability to detect a critical reduction of *N_e_* beyond that of the simulation.

In addition, the temporal variance approach, as well as other tests comparing populations before and after a population reduction (e.g. tests for decreases in heterozygosity, allelic diversity, haplotype and nucleotide diversity, etc.), require that a population be sampled before and at least one generation after the potential bottleneck event. In our study, we collected marine iguana specimens from eleven different islands in 1991 or 1993, before the 1997–1998 El Niño, and in 2004, approximately six years after the end of the warming period. Since we sampled mainly adults and sub-adults in 2004, along with some juveniles, it is possible that some of these individuals were born prior to the El Niño period. However, if a given population was reduced to a critically small size (*N_e_*≤50) during the 1997–1998 warming period, it is very likely that iguanas sampled in 2004 would primarily be individuals born in the first generation after the bottleneck event and would therefore reflect the gene pool of the reduced population. On the other hand, if the population size remained fairly high during the warming period, the gene pool reflected in 2004 would not deviate significantly from its pre-El Niño composition. In the first scenario (*N_e_*≤50), given the number and variability of microsatellite loci employed in this study, we would be able to detect a critical reduction of *N_e_* with a rather high probability as pointed out above. In the second case, however, we would not expect to see any major changes in allele frequencies between time points, since the population makeup had not been significantly altered.

An additional concern is whether the six years following the 1997–1998 El Niño were sufficient for the development of a new generation into adult and sub-adult classes. Female marine iguanas usually reach the age of first reproduction in their fourth or fifth year; however, data from the severe 1982–1983 El Niño show that the age of first reproduction of female marine iguanas may be three years or less following ENSO-induced population crashes [16]. This is partially a result of rapid growth rates that are common during La Niña periods following El Niño [16]. Males, on the other hand, may not gain access to mates until they are at least 12–15 years old, due to strong sexual selection for large-bodied individuals [39]–[40]. However, if a population is far below carrying capacity, which is often the case during strong El Niño events, males can reach maximum size in less than six years, as was found on the island of Santa Fe after the 1982–1983 El Niño [15]. Moreover, large, territorial males appear to suffer the highest rate of mortality during El Niño [15], increasing the likelihood for younger males to occupy mating territories and achieve mating success after strong El Niños. Thus, it is plausible that a new generation could have been established on many islands in the six years following the 1997–1998 El Niño.

### The genetic impact of the 1997–1998 El Niño on Galápagos marine iguanas

There have only been a few attempts to measure the impact of severe El Niño events on genetic diversity in natural populations, with mixed results. Randomly amplified polymorphic DNA (RAPD) markers have shown that heterozygosity in intertidal kelp (*Lessonia nigrescens*) populations along the Chilean coast fell by 50% compared with unaffected populations following the 1982–1983 El Niño [23]. Also, Galápagos penguins (*S. mendiculus*), whose populations have been severely diminished by ENSO, have significantly lower observed heterozygosity compared to the common and unaffected Magellanic penguin (*Spheniscus magellanicus*) from South America, possibly the outcome of a series of strong El Niño events [24]. Conversely, a study on butterflies (*Arhopala epimuta*) in Borneo found that El Niño-induced forest fires did not alter the temporal genetic structure of microsatellite loci (5 loci) or mtDNA (control region) in populations that were sampled before and after the 1997–1998 El Niño [25].

In this study, we focus on the impact of ENSO on multiple populations of Galápagos marine iguanas, which are also known to experience large population reductions (up to 90%) during intense El Niño periods [14]. Although high levels of mortality were recorded on a number of different islands during the 1997–1998 El Niño, the microsatellite data indicate that only one of the 11 populations examined (Marchena) showed signatures of either a critically small population size during the time interval in which the El Niño event occurred or a reduction in genetic diversity following the warming period. Based on the temporal variance approach, there is strong support for a *N_e_* below 50 on Marchena during the period between 1993 and 2004 (Figures 2a and 2b), indicating that the population must have become quite small during this time. F_ST_ values, which are also based on differences in allele frequencies between two samples, support this finding, since a statistically significant level of differentiation between time points was only found for Marchena (Table 1). Lastly, only the population from Marchena exhibited a significant decrease in locus-specific heterozygosity values between the first and second samplings (Table 1 and Table S2).

The results of the mtDNA data were similar, where Marchena exhibited the largest decrease in haplotype diversity and nucleotide diversity from 1993 to 2004 (Figure 2a; Table 2). However, unlike the microsatellite data, the population from Genovesa also showed a significant decrease in Hd between samplings indicating a possible population reduction. Additionally, coalescent simulations showed that observed Hd values were significantly lower than expected under neutral evolution for both Marchena and Genovesa in 2004 but not in the first sampling predating the El Niño (see Table 2).

The genetic signature of a population decline detected between 1991 and 2004 on Genovesa likely reflects the population crash, from 15,000 to 900 individuals, that was observed during the 1997–1998 El Niño event, and there is no evidence for other incidents which may have drastically effected the demography of this island during this time period [14 and Martin Wikelski pers. comm.]. Conversely, for Marchena, marine iguanas may also have been highly impacted by a strong volcanic eruption in 1991, where lava flows continued for at least 40 days. This eruption is likely to have caused mortality of marine iguanas in both the terrestrial environment (as shown for Galápagos tortoises on the island of Isabela [41]) and the marine environment, where water was described as “too hot to touch” and was observed to have killed many fish and other aquatic organisms [42]. Although the actual eruption occurred two years before the initial sampling on Marchena in 1993, a new generation is not likely to have emerged until some point between 1993 and 2004, causing its genetic effects to be confounded with that of the 1997–1998 El Niño. In this case we can imagine three scenarios where populations were reduced due to the eruption, to El Niño, or the combined effect of both. The last is an intriguing option given the magnitude of the population bottleneck on Marchena compared to the other populations.

Additionally, there are two other forces which may have influenced marine iguana populations between 1991/1993 and 2004: the long term, but weaker El Niño period extending from 1990–1995 [1], [11], and an oil spill that occurred off the island of San Cristóbal in 2001 [43]. The former cannot be ruled out, but the genetic impact of the oil spill could not be reflected in our data since the three years between the spill and the 2004 sampling are not sufficient for the establishment of a new generation of marine iguanas, which is an important requirement of the temporal variance method. Thus, this does not rule out the possibility that the oil spill impacted the effective population size of the iguanas, but rather that, with our study design, we are simply not able to detect it.

Under random mating, heterozygosity is lost from a population at a rate of 1/2*N_e_* (1/*N_ef_* in the case of mtDNA) per generation. Thus, genetic diversity is lost more rapidly in small populations. Yet, during brief bottleneck events, only a small amount of the total heterozygosity is lost, even after a considerable reduction in population size (e.g. 2% after the first generation of a bottleneck of *N_e_* = 50) [44]. However, the loss of diversity follows an exponential decay process, where long-term decreases in the number of individuals can have a detrimental impact on *N_e_*. Therefore, it is not surprising that, with the exception of Marchena, a single, strong El Niño event did not stimulate a loss of heterozygosity in the populations, even if mortality rates were high. The temporal variance approach, on the other hand, relies on shifts in allele frequencies rather than loss of diversity, and has been shown to be particularly sensitive in estimating *N_e_* in small populations [28], [45]. Since, as discussed above, our study design offers sufficient statistical power to detect a bottleneck, the large confidence intervals surrounding the majority of *N_e_* estimates suggest that the effective size of most populations of marine iguanas were so large that they underwent only slight shifts in allele frequencies. Such temporal stability has been observed in a number of other studies [46], [47] and is quite plausible given the large census size estimates of marine iguanas recorded of many islands [14].

### Recommendations for future study design in assessing the genetic impact of El Niño

Although we did not find a strong influence of El Niño on genetic diversity in marine iguanas, the data show that the genetic impact of a single, intense environmental challenge may vary even within a single species and depends on the specific history of a population. The low *N_e_* estimate for Marchena, calculated over an interval in which a severe El Niño event and a volcanic eruption occurred, suggests that it is important to consider the potential synergistic relationship between El Niño and other phenomena. While we saw little evidence of a bottleneck in the majority of our populations, future experimental designs must account for multiple natural (e. g. long-term, weaker El Niño periods) and human-induced (e.g. oil spill) disturbances, or else the causes of population reductions may be confounded.

Evolutionary theory predicts that the long-term effective size of a population is the harmonic mean of *N_e_* over many generations [44]. Under this scenario, long-term *N_e_* is heavily influenced by generations in which the effective population size is small. Therefore, the high mortality rates of marine iguanas associated with El Niño warming may compound over multiple ENSO cycles. Since climate models predict that the strength and frequency of El Niño events will continue to increase, a true understanding of the long-term impact of ENSO on population persistence may only come from experiments designed to measure the genetic impact of this phenomenon following a series of consecutive events.

## Supporting Information

Table S1 Sampling localities by island and sample sizes for 13 microsatellite loci and mitochondrial control region sequences (1183 bp) for the two temporal samplings (1991/1993 and 2004). The first column lists sampling localities by island, specific sampling location (in parentheses), and geographical coordinates. Sample sizes for 13 microsatellite loci and mitochondrial control region sequences (1183 bp) are reported in separate columns for the two temporal samplings before the 1997–1998 El Niño (in 1991 or 1993, or both years for Santa Fé) and after the 1997–1998 El Niño in the year 2004.
(0.04 MB DOC)

Table S2 Locus specific heterozygosity values for 13 microsatellite loci for pre- and post-El Niño island samplings. Locus specific heterozygosities calculated using the program ARLEQUIN for marine iguana populations sampled before the 1997–1998 El Niño (in 1991 or in 1993 or as for Santa Fé in both years) and after the 1997–1998 El Niño in the year 2004. The first column (locus) shows names of microsatellite loci. The other columns report the heterozygosity for a given population for each time point. Differences in heterozygosity between time points were tested with a Wilcoxon signed ranks test and associated p-values are provided in the last line. Significant p-values (p<0.05) are marked with an asterik (*). The only significant decrease of locus specific heterozygosity from 1993 to 2004 was found for the population on Marchena, whereas Santa Cruz showed a significant increase during this period.
(0.11 MB DOC)

Table S3 List of haplotype diversity (Hd) values and their variance (V) for marine iguana populations. List of haplotype diversity (Hd) values and their variance (V) for marine iguana populations from both time points. T-values and their corresponding probabilities are based on the test adapted by Nei [37] and reflect whether Hd values are significantly different between time points. Positive t values reflect a decrease Hd from the first to second sampling, while negative t values reflect an increase.
(0.04 MB DOC)

Table S4 List of nucleotide diversity (π) values and their variance (V) for marine iguana populations. List of nucleotide diversity (π) values and their variance (V) for marine iguana populations from both time points. T-values and their corresponding probabilities are based on the test adapted by Nei [37] and reflect whether π values are significantly different between time points. Positive t values reflect a decrease in π from the first to the second sampling, while negative t values reflect an increase.
(0.04 MB DOC)

Figure S1 Graphical display of the TM3 results (estimates of the effective population size [Ne] generated from the temporal variance of allele frequencies using a likelihood-based approach) as provided by the program NeESTIMATOR. Graphical display of the TM3 results (estimates of the effective population size (Ne) generated from the temporal variance of allele frequencies using a likelihood-based approach) as provided by the program NeESTIMATOR (see Methods). Point estimates for different values of Ne are shown (x-axis) with their corresponding log likelihood values (y-axis). For each island, the estimated Ne with the highest log likelihood, the calculated 95% confidence interval (CI), and the upper bound of the estimate are provided below the graphic.
(2.03 MB DOC)
