# Supplementary material for: Genetic Impact of a Severe El Niño Event on Galápagos Marine Iguanas (Amblyrhynchus cristatus)
Source: PLoS One. 2007 Dec 12;2(12):e1285. doi: 10.1371/journal.pone.0001285 (PMC2110882; doi:10.1371/journal.pone.0001285)
Supplement: Table S1 — Sampling localities by island and sample sizes for 13 microsatellite loci and mitochondrial control region sequences (1183 bp) for the two temporal samplings (1991/1993 and 2004). The first column lists sampling localities by island, specific sampling location (in parentheses), and geographical coordinates. Sample sizes for 13 microsatellite loci and mitochondrial control region sequences (1183 bp) are reported in separate columns for the two temporal samplings before the 1997–1998 El Niño (in 1991 or 1993, or both years for Santa Fé) and after the 1997–1998 El Niño in the year 2004. (0.04 MB DOC) [file pone.0001285.s001.doc]

| Island (Sampling Location) | Samples collected before the 1997-1998 El Niño | Samples collected after the 1997-1998 El Niño (2004) |
| --- | --- | --- |
|  | Microsatellites / MtDNA | Microsatellites / MtDNA |
| Fernandina (Punta Mangle) 91.38926°W 0.44289°S | 29/28  (1993) | 49/45 |
| Isabela (Punta Albemarle) 91.37809°W 0.13988°N | 30/26  (1993) | 31/28 |
| Santa Cruz (Camaño) 90.27856°W 0.75907°S | 36/35  (1991) | 30/28 |
| Santiago (James Bay)  90.86495°W 0.24215°S | 23/32  (1993) | 28/40 |
| Pinta (Caleta Ibetson)  90.73530°W 0.54586°N | 41/41  (1993) | 53/53 |
| Marchena (Bahia Negra)  90.50726°W 0.29429°N | 30/29  (1993) | 49/49 |
| Genovesa (Campamente)  89.97349°W 0.31065°N | 54/46  (1991) | 38/35 |
| Santa Fé (Miedo)  90.03333°W 0.83333°S | 37/37  (1991/93) | 45/45 |
| Floreana (Punta Montura)  90.49815°W 01.29700°S | 20/30  (1993) | 31/30 |
| Española (Punta Cevallos)  89.62033°W 01.38819°S | 40/40  (1993) | 30/58 |
| San Cristóbal (Lobería)  89.61793°W 0.92256°S | 30/31  (1993) | 52/52 |
