# Supplementary material for: Genetic Impact of a Severe El Niño Event on Galápagos Marine Iguanas (Amblyrhynchus cristatus)
Source: PLoS One. 2007 Dec 12;2(12):e1285. doi: 10.1371/journal.pone.0001285 (PMC2110882; doi:10.1371/journal.pone.0001285)
Supplement: Table S2 — Locus specific heterozygosity values for 13 microsatellite loci for pre- and post-El Niño island samplings. Locus specific heterozygosities calculated using the program ARLEQUIN for marine iguana populations sampled before the 1997–1998 El Niño (in 1991 or in 1993 or as for Santa Fé in both years) and after the 1997–1998 El Niño in the year 2004. The first column (locus) shows names of microsatellite loci. The other columns report the heterozygosity for a given population for each time point. Differences in heterozygosity between time points were tested with a Wilcoxon signed ranks test and associated p-values are provided in the last line. Significant p-values (p<0.05) are marked with an asterik (*). The only significant decrease of locus specific heterozygosity from 1993 to 2004 was found for the population on Marchena, whereas Santa Cruz showed a significant increase during this period. (0.11 MB DOC) [file pone.0001285.s002.doc]

| Locus | Fernadina | | **San Cristóbal** | | **Floreana** | | **Genovesa** | | **Marchena** | | **Pinta** | |
| --- | --- | --- | --- | --- | --- | --- | --- | --- | --- | --- | --- | --- |
|  | **1993** | **2004** | **1993** | **2004** | **1993** | **2004** | **1991** | **2004** | **1993** | **2004** | **1993** | **2004** |
| MIGE2 | 0.853 | 0.851 | 0.779 | 0.786 | 0.799 | 0.783 | 0.646 | 0.748 | 0.834 | 0.782 | 0.645 | 0.732 |
| MIG-E3 | 0.882 | 0.887 | 0.683 | 0.683 | 0.883 | 0.824 | 0.778 | 0.738 | 0.786 | 0.752 | 0.708 | 0.684 |
| MIG-E4 | 0.898 | 0.878 | 0.829 | 0.844 | 0.882 | 0.911 | 0.735 | 0.755 | 0.762 | 0.829 | 0.69 | 0.72 |
| MIG-E6 | 0.85 | 0.845 | 0.691 | 0.689 | 0.512 | 0.669 | 0.68 | 0.598 | 0.815 | 0.793 | 0.755 | 0.763 |
| MIG-E8 | 0.812 | 0.853 | 0.732 | 0.703 | 0.848 | 0.801 | 0.811 | 0.841 | 0.877 | 0.875 | 0.716 | 0.737 |
| MIG-E10 | 0.862 | 0.869 | 0.782 | 0.791 | 0.897 | 0.86 | 0.64 | 0.635 | 0.81 | 0.826 | 0.739 | 0.726 |
| MIG-E11 | 0.843 | 0.853 | 0.751 | 0.755 | 0.795 | 0.821 | 0.808 | 0.804 | 0.87 | 0.853 | 0.613 | 0.631 |
| MIG-E12 | 0.873 | 0.903 | 0.798 | 0.802 | 0.814 | 0.826 | 0.764 | 0.707 | 0.826 | 0.79 | 0.396 | 0.242 |
| MIG-E13 | 0.865 | 0.852 | 0.732 | 0.734 | 0.769 | 0.763 | 0.659 | 0.641 | 0.758 | 0.722 | 0.712 | 0.698 |
| MIG-E14 | 0.924 | 0.929 | 0.835 | 0.835 | 0.933 | 0.943 | 0.734 | 0.763 | 0.887 | 0.866 | 0.709 | 0.561 |
| MIG-E15 | 0.713 | 0.736 | 0.591 | 0.508 | 0.847 | 0.829 | 0.647 | 0.67 | 0.759 | 0.704 | 0.77 | 0.682 |
| MIG-E16 | 0.764 | 0.773 | 0.495 | 0.544 | 0.722 | 0.745 | 0.731 | 0.781 | 0.714 | 0.713 | 0.522 | 0.441 |
| MIG-E17 | 0.598 | 0.686 | 0.561 | 0.662 | 0.798 | 0.848 | 0.665 | 0.721 | 0.74 | 0.643 | 0.673 | 0.663 |
| Diff. P | **0.094** | | **0.278** | | **0.787** | | **0.588** | | **0.032*** | | **0.305** | |
|  |  | |  | |  | |  | |  | |  | |
| Locus | Santiago | | **Santa Cruz** | | **Española** | | **Isabela** | | **Santa Fé** | |  | |
|  | **1993** | **2004** | **1991** | **2004** | **1993** | **2004** | **1993** | **2004** | **1991/93** | **2004** |  | |
| MIGE2 | 0.804 | 0.82 | 0.697 | 0.779 | 0.872 | 0.852 | 0.812 | 0.811 | 0.768 | 0.743 |  | |
| MIG-E3 | 0.821 | 0.799 | 0.868 | 0.878 | 0.662 | 0.719 | 0.867 | 0.847 | 0.8 | 0.853 |  | |
| MIG-E4 | 0.778 | 0.775 | 0.878 | 0.892 | 0.891 | 0.887 | 0.863 | 0.878 | 0.876 | 0.846 |  | |
| MIG-E6 | 0.662 | 0.704 | 0.831 | 0.86 | 0.613 | 0.565 | 0.827 | 0.811 | 0.832 | 0.826 |  | |
| MIG-E8 | 0.745 | 0.734 | 0.839 | 0.825 | 0.837 | 0.798 | 0.855 | 0.816 | 0.83 | 0.804 |  | |
| MIG-E10 | 0.817 | 0.861 | 0.842 | 0.845 | 0.86 | 0.854 | 0.783 | 0.8 | 0.86 | 0.89 |  | |
| MIG-E11 | 0.816 | 0.792 | 0.761 | 0.763 | 0.791 | 0.781 | 0.794 | 0.798 | 0.725 | 0.666 |  | |
| MIG-E12 | 0.818 | 0.782 | 0.87 | 0.917 | 0.808 | 0.851 | 0.88 | 0.885 | 0.802 | 0.854 |  | |
| MIG-E13 | 0.729 | 0.791 | 0.782 | 0.815 | 0.73 | 0.768 | 0.814 | 0.854 | 0.577 | 0.612 |  | |
| MIG-E14 | 0.908 | 0.912 | 0.881 | 0.901 | 0.905 | 0.939 | 0.919 | 0.925 | 0.907 | 0.913 |  | |
| MIG-E15 | 0.843 | 0.853 | 0.888 | 0.884 | 0.748 | 0.707 | 0.608 | 0.575 | 0.562 | 0.553 |  | |
| MIG-E16 | 0.668 | 0.65 | 0.687 | 0.759 | 0.792 | 0.846 | 0.769 | 0.74 | 0.542 | 0.55 |  | |
| MIG-E17 | 0.64 | 0.484 | 0.675 | 0.66 | 0.804 | 0.796 | 0.7 | 0.727 | 0.847 | 0.843 |  | |
| Diff. P | **0.893** | | **0.04*** | | **0.893** | | **0.893** | | **0.893** | |  | |
