# Supplementary material for: Genetic Impact of a Severe El Niño Event on Galápagos Marine Iguanas (Amblyrhynchus cristatus)
Source: PLoS One. 2007 Dec 12;2(12):e1285. doi: 10.1371/journal.pone.0001285 (PMC2110882; doi:10.1371/journal.pone.0001285)
Supplement: Table S3 — List of haplotype diversity (Hd) values and their variance (V) for marine iguana populations. List of haplotype diversity (Hd) values and their variance (V) for marine iguana populations from both time points. T-values and their corresponding probabilities are based on the test adapted by Nei [37] and reflect whether Hd values are significantly different between time points. Positive t values reflect a decrease Hd from the first to second sampling, while negative t values reflect an increase. (0.04 MB DOC) [file pone.0001285.s003.doc]

| Location | Hd 1991/93 | *V*(Hd) 1991/93 | Hd 2004 | *V*(Hd) 2004 | *t* value | df | P<0.025  Upper Tail |
| --- | --- | --- | --- | --- | --- | --- | --- |
| Española | 0.69487 | 0.00247 | 0.60799 | 0.00278 | 1.199 | 96 | No |
| Floreana | 0.64598 | 0.00696 | 0.85517 | 0.00164 | -2.256 | 58 | Yes |
| Fernandina | 0.91534 | 0.00190 | 0.94949 | 0.00027 | -0.733 | 71 | No |
| Genovesa | 0.66377 | 0.00318 | 0.43193 | 0.00891 | 2.109 | 79 | Yes |
| Isabela | 0.75077 | 0.00333 | 0.62169 | 0.00649 | 1.303 | 52 | No |
| Marchena | 0.68473 | 0.00333 | 0.25000 | 0.00515 | 4.721 | 76 | Yes |
| Pinta | 0.36951 | 0.00708 | 0.48113 | 0.00525 | -1.005 | 92 | No |
| Santiago | 0.57460 | 0.00326 | 0.50128 | 0.00086 | 1.142 | 70 | No |
| Santa Fé | 0.72823 | 0.00143 | 0.68990 | 0.00057 | 0.857 | 80 | No |
| San Cristóbal | 0.65376 | 0.00164 | 0.59804 | 0.00223 | 0.896 | 81 | No |
