# Supplementary material for: Genetic Impact of a Severe El Niño Event on Galápagos Marine Iguanas (Amblyrhynchus cristatus)
Source: PLoS One. 2007 Dec 12;2(12):e1285. doi: 10.1371/journal.pone.0001285 (PMC2110882; doi:10.1371/journal.pone.0001285)
Supplement: Table S4 — List of nucleotide diversity (π) values and their variance (V) for marine iguana populations. List of nucleotide diversity (π) values and their variance (V) for marine iguana populations from both time points. T-values and their corresponding probabilities are based on the test adapted by Nei [37] and reflect whether π values are significantly different between time points. Positive t values reflect a decrease in π from the first to the second sampling, while negative t values reflect an increase. (0.04 MB DOC) [file pone.0001285.s004.doc]

| Location | π 1991/93 | *V*(π) 1991/93 | π 2004 | *V*(π) 2004 | *t* value | df | P<0.025  Upper Tail |
| --- | --- | --- | --- | --- | --- | --- | --- |
| Española | 0.00248 | 0.0000003600 | 0.00153 | 0.0000000100 | 1.562 | 96 | No |
| Floreana | 0.00396 | 0.0000002809 | 0.00435 | 0.0000001600 | -0.587 | 58 | No |
| Fernandina | 0.00317 | 0.0000000576 | 0.00331 | 0.0000001521 | -0.306 | 71 | No |
| Genovesa | 0.00323 | 0.0000007056 | 0.00268 | 0.0000009604 | 0.426 | 79 | No |
| Isabela | 0.00351 | 0.0000004356 | 0.00331 | 0.0000003600 | 0.224 | 52 | No |
| Marchena | 0.00323 | 0.0000002209 | 0.00212 | 0.0000003721 | 1.441 | 76 | No |
| Pinta | 0.00108 | 0.0000000676 | 0.00127 | 0.0000000484 | -0.558 | 92 | No |
| Santiago | 0.00574 | 0.0000003600 | 0.00594 | 0.0000001225 | -0.288 | 70 | No |
| Santa Fé | 0.00275 | 0.0000000576 | 0.00293 | 0.0000000324 | -0.600 | 80 | No |
| San Cristóbal | 0.00234 | 0.0000000144 | 0.00212 | 0.0000000289 | 1.057 | 81 | No |
