# Supplementary material for: Genetic Impact of a Severe El Niño Event on Galápagos Marine Iguanas (Amblyrhynchus cristatus)
Source: PLoS One. 2007 Dec 12;2(12):e1285. doi: 10.1371/journal.pone.0001285 (PMC2110882; doi:10.1371/journal.pone.0001285)
Supplement: Figure S1 — Graphical display of the TM3 results (estimates of the effective population size [Ne] generated from the temporal variance of allele frequencies using a likelihood-based approach) as provided by the program NeESTIMATOR. Graphical display of the TM3 results (estimates of the effective population size (Ne) generated from the temporal variance of allele frequencies using a likelihood-based approach) as provided by the program NeESTIMATOR (see Methods). Point estimates for different values of Ne are shown (x-axis) with their corresponding log likelihood values (y-axis). For each island, the estimated Ne with the highest log likelihood, the calculated 95% confidence interval (CI), and the upper bound of the estimate are provided below the graphic. (2.03 MB DOC) [file pone.0001285.s005.doc]

(A) Fernandina *N_e_*=116,130 (CI = 298 – 120,000); upper bound *N_e_*=120,000

(B) San Cristóbal *N_e_*=960 (CI = 73 – 1,000); upper bound *N_e_*=1,000

(C) Floreana *N_e_*=770 (CI = 0 – 16,000); upper bound *N_e_*=16,000

(D) Genovesa *N_e_*=783 (CI = 0 – 15,000); upper bound *N_e_*=15,000

(E) Marchena *N_e_*=40 (CI = 21 – 86); upper bound *N_e_*=1000

(F) Pinta *N_e_*=2,036 (CI = 0– 6000); upper bound *N_e_*=6000

(G) Santiago *N_e_*=3,657 (CI = 18 – 4000); upper bound *N_e_*= 4000

(H) Santa Cruz (Camaño) *N_e_*=12,011 (CI = 59 – 13,000); upper bound *N_e_*=13,000

(I) Española *N_e_*=18,120 (CI = 0 – 21,000); upper bound *N_e_*=21,000

(J) Isabela *N_e_*=37,523 (CI = 0 – 40,000); upper bound *N_e_*=40,000

(K) Santa Fé *N_e_*=15,006 (CI = 409 – 16,000); upper bound *N_e_*=16,000
